# Supplementary material for: Bone marrow derived mast cells injected into the osteoarthritic knee joints of mice induced by sodium monoiodoacetate enhanced spontaneous pain through activation of PAR2 and action of extracellular ATP
Source: PLoS One. 2021 Jun 4;16(6):e0252590. doi: 10.1371/journal.pone.0252590 (PMC8177436; doi:10.1371/journal.pone.0252590)
Supplement: Attachment — Submitted filename: Response to Reviewers.docx [file pone.0252590.s001.docx]

Dear Dr. Rosanna Di Paola
Academic Editor
PLOS ONE

Thank you for your E-mail of March 9, 2021 about our manuscript entitled "Bone marrow derived mast cells injected into the osteoarthritic knee joints of mice induced by sodium monoiodoacetate enhanced spontaneous pain through activation of PAR2 and action of extracellular ATP." (PONE-D-21-01925). I have amended our manuscript as far as possible in light of the opinion of the reviewers. To answer the question raised by the reviewer, we revised the manuscript.

Answer to the reviewer

For reviewer #1:

1) Some descriptions in Abstract (Line 26 and Line 33-36), Introduction (Line 55-57) and Discussion (Line 360, Line 366, Line 374 and Line 380) were modified for clarity in English.

2) In Fig 3, deterioration of superficial cartilage detected by toluidine blue staining was evident in mice belonging to A, B, E and F groups; however, significant difference in the degree of the deterioration was not observed among these groups. These results may be related to the clinical observation that OA pain does not necessarily correlate with the extent of joint degeneration. We added such description in Discussion (Line 398-402).

3) In the preliminary experiments, we have also observed stance score on the basis of the previously described methods (Heilborn U, Berge OG, Arborelius L, Brodin E. Spontaneous nociceptive behaviour in female mice with Freund's complete adjuvant- and carrageenan-induced monoarthritis. Brain Res. 2007 Apr 27;1143:143-9. doi: 10.1016/j.brainres.2007.01.054), and obtained basically the similar results to those from single paw standing; however, we adopted single paw standing for quantification of pain in this paper because single paw standing gave more quantitative data than stance score.

4) Our experiments clearly indicate that PAR2 and ATP are involved in the generation of spontaneous pain in OA; however, many other factors and different types of cells and tissues are possibly involved in elicitation of pain. We are concerned about too much simplification of the mechanistic aspect of OA pain by drawing a diagram that summarize our working hypothesis;

therefore, we would like to omit the diagram that the reviewer has suggested.

For reviewer #2:

a) We described conclusion in the newly inserted section of Conclusion as follow: “Bone marrow derived mast cells (BMMCs) injected into the MIA-induced OA knee joints enhanced spontaneous pain, but these cells showed no pain-enhancing effects when injected into the knee joints of control mice that had not been treated with MIA. PAR2 and extracellular ATP were possibly involved in the pain-enhancing effects of BMMCs. “ (Line 430-434).

b) We used enough mice to obtain significant results. Number of mice used for each experiment was described in the legends of Fig 4, Fig 6, Fig 7 and Fig 10.

c) In introduction we added one paper about the effects of a pharmaceutical agent on the pain elicited in MIA-induced OA (Ref 28) (Line 70).

We found after submission that Ref 49 was the same as Ref 27, so we deleted Ref 49. Accordingly Ref number was modified. We modified reference format to fit it to the PLOS ONE duideline.

I believe that modification of the manuscript made this paper more correct and better. I would like to thank the reviewers for their kind comments. I hope this revised paper is now suitable for publication in the PLOS ONE.

Sincerely yours,

Osami Habuchi, Ph.D.
